# Impact of Sarcopenia on Functional and Oncological Outcomes After Radical Prostatectomy

**DOI:** 10.3389/fsurg.2020.620714

**Published:** 2021-02-03

**Authors:** Markus Angerer, Georg Salomon, Dirk Beyersdorff, Margit Fisch, Markus Graefen, Clemens M. Rosenbaum

**Affiliations:** ^1^Martini-Klinik Prostate Cancer Center, University Hospital Hamburg-Eppendorf, Hamburg, Germany; ^2^Department of Urology, University Hospital Hamburg-Eppendorf, Hamburg, Germany; ^3^Department of Radiology, Interventional Radiology and Nuclear Medicine, University Hospital Hamburg-Eppendorf, Hamburg, Germany; ^4^Department of Urology, Asklepios Hospital Barmbek, Hamburg, Germany

**Keywords:** prostate cancer, sarcopenia, radical prostatectomy, oncological outcome, functional outcome

## Abstract

**Introduction and Objectives:** Knowledge about the significance of sarcopenia (muscle loss) in prostate cancer (PCa) patients is limited. The aim of this study was to determine the influence of skeletal muscle index (SMI) on early functional and pathological outcome in patients undergoing radical prostatectomy (RP).

**Materials and Methods:** One hundred randomly chosen patients who received RP between November 2016 and April 2017 at Martini-Klinik (Hamburg, Germany) were retrospectively assessed. SMI (skeletal muscle mass cross-sectional area at L3/m^2^) was measured by preoperative staging computed tomography scans at L3 level. Cox regression analysis was applied to determine the impact of SMI on post-operative outcome. Follow-up was 12 months. Continence was defined as no more than one safety pad per day.

**Results:** Mean age of the cohort was 63.6 years. Mean SMI was 54.06 cm^2^/m^2^ (range, 40.65–74.58 cm^2^/m^2^). Of the patients, 41.4% had pT2, 28.7% had pT3a, and 29.9% had pT3b or pT4 PCa. SMI revealed to be without significant correlation on tumor stage. Follow-up data of 55 patients were available for early functional outcome analysis. SMI showed no significant influence on erectile function in multivariable Cox regression analysis. In multivariable Cox regression analysis, SMI turned out to have no influence on continence rates 6 weeks after surgery.

**Conclusion:** The present study shows that patients undergoing RP have a wide range of SMI. Unlike in other urological malignancies, there was no significant impact of SMI on early functional outcome and pathological outcome. A larger cohort is needed to confirm these results.

## Introduction

Prostate cancer (PCa) is the most common cancer and the third most common cause of cancer death among men in the western world (1). According to the German health report in corporation with the Robert-Koch-Institute, ~49,000 cases of PCa are reported per annum; the incidence is 120 in all age classes in Germany (2).

Radical prostatectomy (RP), brachytherapy (BT), and the advanced technique of radiation using intensity-modulated radiation therapy (IMRT) are the three most common treatment procedures for localized prostate cancer. All techniques show no significant contrariness in overall survival (3, 4). RP embodies one of the most often used treatment option in localized prostate cancer, mainly implemented as either retropubic open RP or laparoscopic/robot-assisted RP (5).

The most recognized risk factors for developing PCa are increasing age, ethnic origin, and family history (6). The familiar predisposition suggests an inherited genetic component to PCa (7, 8). Preoperative prostate-specific antigen (PSA), pathological stage, Gleason score, and surgical margins status predicted BCR after RP (9).

“Sarcopenia is a progressive and generalized skeletal muscle disorder that is associated with increased likelihood of adverse outcomes including falls, fractures, physical disability, and mortality” as defined by the European Working Group on Sarcopenia in Older People (EWGSOP) (10). Sarcopenia is increasingly recognized as a risk factor for a worse performance especially in patients suffering from a malignant tumor disease (11). Lately, the presence of sarcopenia has been identified as a “prognostic marker of disease recurrence, cancer-specific mortality (CCM), and all-cause mortality (ACM)” in patients with not particularly urological malignancies but also, e.g., gynecological and gastrointestinal cancer diseases (12–15).

The definition of sarcopenia is based on the skeletal muscle index (SMI). The muscle volume can reproducibly be measured by computed tomography (CT) or magnetic resonance imaging (MRI) (10).

Among others, potential risk factors of perioperative complications are BMI >30 and Charlson comorbidity index (CCI) ≥1 (16). Performance status and comorbidity are generally subjective and difficult to define. The American Association of Anesthesiologists (ASA) score, the Eastern Cooperative Oncology Group (ECOG) performance status, and CCI are commonly calculated prognostic factors for analyzing post-operative outcomes. Yet, they have been doubted to identify those patients at highest risk of perioperative morbidity and mortality, despite the successfully recognition of all status (14, 17). Sarcopenic patients have been demonstrating a higher rate of perioperative complications (18–21).

This resulted to proclaim sarcopenia as an important acknowledging factor in treatment planning, decision-making, and gaining information regarding patients peri- and post-operative outcome (17).

In men diagnosed with prostate cancer, little is known about the role of sarcopenia influencing the functional and oncological outcome. One study concluded that sarcopenia does not predict the oncological outcome after RP (22). Another study that investigated men undergoing radiotherapy for PCa identified a significant impact of skeletal muscle reduction on non-cancer mortality (23).

We hypothesized that sarcopenia may be correlate with a higher complication rate and worse oncological outcome in men undergoing RP. Consequently, we examined the association between sarcopenia and perioperative as well as oncological outcome in men undergoing RP (17).

## Materials and Methods

We retrospectively analyzed 100 patients who were treated with RP, either open retropubic RP or laparoscopic, robot-assisted RP at a high-volume center (Martini-Klinik Prostate Cancer Center, Hamburg-Eppendorf, Germany) between November 2016 and April 2017. RP was only performed consistently by eight highly trained surgeons performing RRP and robot-assisted RP regularly.

We have identified the patients randomly within our database. Staging CT scans were obtained by patients with intermediate- and high-risk PCa defined by D'Amico as a clinical T stage ≥cT2c, a Gleason score ≥8, or a PSA >20 ng/ml (24).

CT images were obtained from the patients preoperative CT scans of the abdomen or pelvis. Included were only patients with sufficient quality of CT images. Patients' informed consent for data collection was obtained. The cross-sectional area of all skeletal muscle at third lumbar vertebrae 3 (L3) has a high correlation to the body's general muscle volume (18). Lumbal SMI is calculated by the cross-sectional area of all skeletal muscle at L3 by height squared (m^2^) and reported as cm^2^/m^2^. Clinical, blood sample results, and oncological data were collected from the hospitals' documenting program, Soarian, and Martini Data Registry.

**Figure 1:**
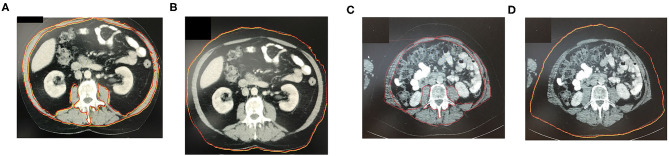
**(A,B)** Axial CT-image at L3 vertebra depicting patient without sarcopenia. **(C,D)** Axial CT-image at L3 vertebra depicting patient with BMI and significantly different SMI (sarcopenic patient). The red marked area represents the cross-sectional area of all skeletal muscle at L3 including the rectus abdominus; internal, external, and lateral obliques; psoas; quadratus lumborum; and erector spinae muscles. The red marked line in the image represents patients abdominal circumference.

A single axial image at the level of L3 was selected, and the cross-sectional area of all skeletal muscle at L3 was measured after identifying the muscle-specific attenuation thresholds (−29–150 HU). For the measurement, musculus rectus abdominus; internal, external, and lateral musculus obliquus abdominis; musculus psoas; musculus quadratus lumborum; and musculus erector spinae were included. Axial CT images at L3 vertebra depicting patient without sarcopenia are shown in Figures 1A,B as compared to patients with different BMIs and significantly different SMI shown in Figures 1C,D. The radiologist program Centricty Viewer GE was used for image analysis. Image analysis was performed by the same investigator who was unaware about the patients' cancer-specific data.

Clinical and pathological data were collected. Clinical data are include information on age, clinical TNM classification (clinical tumor and lymph node stage), preoperative PSA, continence by the number of pad usage per day, as well as preoperative androgen deprivation therapy. Pathological data collected included prostate biopsy Gleason score, pathological specimen Gleason score, pathological TN classification (pathological tumor and lymph node stage), and surgical margin status.

Taking into consideration the EWGSOP definition of sarcopenia, SMI was based on sex- and BMI-specific cutoffs for men <43 cm^2^/m^2^ (BMI <25) and <53 cm^2^/m^2^ (BMI >25) to classify patients as sarcopenic vs. non-sarcopenic (25).

Urine continence was defined not to use more than 1 safety Pad per Day.

### Statistical Analysis

Clinical and pathological variables were compared between the sarcopenic and non-sarcopenic patients. Age, BMI (in kg/m^2^), pathological tumor and lymph node stage, pathological surgical margin status, PSA, and Gleason score are taken into account for comparison of the two groups. Continuous features were summarized with medians and interquartile ranges (IQRs). Categorical features were summarized with frequency counts and percentages and compared using the chi-square test. The primary interest was to evaluate the functional and oncological outcome.

Logistic regression analysis was used to estimate the oncological outcome and biochemical recurrence (BCR). BCR was defined as PSA value >0.2 ng/ml after RP. Urine continence was assessed by univariable and multivariable Cox proportional hazards regression models and summarized with hazards ratios (HRs) and 95% confidence intervals (95% CIs).

Furthermore, statistically significant prognosticators on univariable analysis were also analyzed in multivariable models. A *p* < 0.05 was considered to be statistically significant.

For follow-up assessment, patients were evaluated for urinary continence and erectile function (EF) after 6 weeks and 12 months after RP. Patient-reported outcomes were registered by standardized Martini–Klinik questionnaires (5).

## Results

We included for the first analysis 99 patients from our database who fulfilled the inclusion criteria. All of them were operated between November 2016 and April 2017. One patient was excluded due to missing data.

SMI measurements of all 99 patients were conducted based on SMI definition; 26 patients (26.3%) were classified as sarcopenic. Descriptive pathological and perioperative characteristics are shown in Table 1.

**Table 1:** Descriptive pathologic and perioperative characteristics of PCa patients that underwent RP between November 2016 and April 2017.

**Characteristics**	**Overall**	**Non-sarcopenic patients**	**Sarcopenic patients**	***p*-value**
No. of patients, *n* (%)	99	73 (73.7)	26 (26.3)	
Age at RP (years), median	65 (59–68.7)	64 (57–67)	68 (61–71)	0.02
Prostate volume (ml), median	39 (30–47.5)	38 (28–48)	41 (33–46)	0.19
SMI (cm^2^/m^2^), median	54 (49.4–58.6)	57 (53–61)	50 (46–50)	<0.001
Nerve-sparing (%), *n*				
Yes	85	62 (84.9)	23 (88.5)	0.91
No	14	11 (15.1)	3 (11.5)	
pT-Stadium (%), *n*				0.81
pT2	42	32 (43.8)	10 (38.5)	
pT3/4	57	41 (56.2)	16 (61.5)	
pN-Status (%), *n*				0.29
Nx/N0	66	46 (63)	20 (67.9)	
N+	33	27 (37)	6 (23.1)	
Gleason (%), *n*				0.35
3 + 3/3 + 4	40	27 (37)	13 (50)	
4 + 4/>4 + 4	59	46 (63)	13 (50)	

Overall, sarcopenic patients were older than non-sarcopenic patients (mean age, 68.0 vs. 64 years, *p* = 0.02). There was no difference between sarcopenic and non-sarcopenic patients in local and lymphonodal pathologic stage or Gleason score. There was no significant difference between sarcopenic and non-sarcopenic patients regarding nerve-sparring surgery (84.9 vs. 88.5%, *p* = 0.91).

In addition, there was no significant difference in urine continence at 1 year after surgery between sarcopenic and non-sarcopenic patients in multivariable logistic regression analysis [odd's ratio (OR), 1.05; 95% confidence interval (CI), 0.96–1.16; *p* = 0.26].

Results are shown in Table 2.

**Table 2:** Urine continence at 1 year after RP.

**Characteristics**	**Odd's ratio**	**95% CI**	***p*-value**
Age at RP	1.05	0.95–1.17	0.31
Prostate volume	1.02	0.98–1.06	0.4
Nerve-sparing			
Yes	Reference		
No	0.38	0.03–3.35	0.4
pT-Stadium			
pT2	Reference		
pT3a	0.53	0.1–2.46	0.43
pT3b/pT4a	1.99	0.35–10.92	0.42
SMI	1.05	0.96–1.16	0.26

In Cox regression analysis, the incidence of BCR did not differ significantly 1 year after surgery between sarcopenic and non-sarcopenic patients [hazard ratio (HR), 0.97; 95% CI, 0.3–3.08; *p* = 0.953].

## Discussion

Sarcopenia represents “a response to both nutrient deprivation and systemic stress, resulting in critical anatomic and functional deficits” (17). Sarcopenia is a major public health issue. Using the definition with highest prevalence estimates, the number of individuals with sarcopenia would rise from 19,740 million in 2016 to 32,338 million in 2045 only in Europe, corresponding to an increase from 20.2 to 22.3% (26).

In this current study, we examined the association between sarcopenia and functional and oncological outcome after RP. Our hypothesis that sarcopenia significantly effects functional and oncologic outcome in men undergoing RP could not be proven.

We noted several findings of interest. First, we determined that in this cohort of patients with RP, 26.3% of patients were classified as sarcopenic preoperatively. The median age of sarcopenic patients was significantly older.

The correlation between BMI and outcome after RP has been investigated often in past. An increase in BMI showed a significant increase risk of peri- and post-operative complications; prolonged operative time, increased blood loss, increased open conversions, longer hospitalization, and higher positive surgical margin rate (27). BMI has known associations with diabetes, coronary artery disease, and hypertension (27). Obesity also has a significant impact on mortality in cancer patients (24). Freedland et al. concluded that elevated BMI has been associated with biochemical failure after radical prostatectomy, due to inferior surgery, which caused a higher rate of positive surgical margin. Also in their cohort, obese men after RP showed worse outcomes, suggesting that obesity may be associated with a biologically more aggressive form of prostate cancer (28–30). Still, it remains controversial regarding the effect on BCR (22).

As mentioned before, McDonald et al. assessed in their study the cross-sectional area at the L4–5 level after radiotherapy for localized prostate cancer retrospective of 653 men (23). They were concluding that sarcopenia significantly increased risk of non-cancer mortality after radiotherapy. Analyzing their cohort, the conclusion is due to the fact that cross-sectional area of all total skeletal muscle was measured at L4–5 and relatively few patients. Furthermore, this study had muscle L4–5 values below the sarcopenic threshold.

Mason et al. published in June 2018 the association between sarcopenia and oncological outcome after RP in a cohort of totally 698 patients and 310 patients identified as sarcopenic (22). They concluded that sarcopenia has no significant association with either perioperative complications or oncological outcome after RP. This study showed a representative number of patients classified sarcopenic (55.6%). Furthermore, there were no significant differences in clinical T or N stage or biopsy Gleason score.

Two different cohort of men with prostate cancer showed contradictory associations of sarcopenia. This may be because of the different populations or different cancer-specific criteria. Patients for RP selected by urologists favoring patients younger in age with a longer life expectancy and reduced comorbidities.

Our data reveal that SMI has neither significant influence on pathological outcome nor on BCR rates after RP.

Furthermore, SMI had no impact on post-operative urine continence in our cohort. These results may suggest that sarcopenia is not a prognostic marker for functional and oncological outcome after RP.

In our study, we acknowledge several limitations to this study. First, we cannot rule out a bias due to random selection of included patients. Not all patients between the period of November 2016 and April 2017 who underwent RP have been selected for analysis and follow-up. Exclusion was caused by missing CT scans, either not readable, poor quality for analysis or missing import; or low-risk PCa patients accordingly to D'Amico classification, which have not received a preoperative CT staging. Another major limitation is that our cohort only figured 99 patients. Therefore, additional subanalyses of risk classifications are necessary. SMI was only measured by preoperative scans. The change in SMI is not considered. Ha et al. showed a significant change in sarcopenia and SMI 1 year after radical cystectomy and might be an effective marker for oncological outcome (31). Another limitation of this study is the time of follow-up after RP, which limits the statement of sarcopenia effecting BCR. The results currently show the 12 months questionnaire feedback. The effect of BCR cannot safely be clarified; hence, the follow-up time must be prolonged. We are continuing to assess follow-up data.

Nevertheless, little is known about the association of sarcopenia on functional and oncological outcome after RP. Our study presents that sarcopenia is not significantly associated with influencing the oncological outcome, urine continence, or BCR after RP.

## Conclusion

Sarcopenia was not significantly associated with worse functional and oncological outcome after RP. In addition, sarcopenia has no significant effect on BCR. Thus, sarcopenia is not a prognostic marker for patients with prostate cancer after RP.

## Data Availability Statement

The raw data supporting the conclusions of this article will be made available by the authors, without undue reservation.

## Ethics Statement

The studies involving human participants were reviewed and approved by Ethikkommission Hamburg. The patients/participants provided their written informed consent to participate in this study.

## Author Contributions

MA was responsible for conceiving the presented idea, designed the study, developed the theory, and performed the computations, literature research, data collection, writing of the manuscript with the help of CR, CT image analysis, and statistical calculations. GS contributed with literature research, investigated, and supervised the findings of this work, helped with manuscript correction and revision. DB contributed with the help of CT image analysis and providing the software, also helped with manuscript correction and revision. MF contributed with the help of manuscript correction and revision. MG contributed with literature, verified the analytical methods, helped with manuscript correction and revision. CR contributed with planning and supervision of the work, helped with statistical calculations, manuscript correction and revision. All authors discussed the results and contributed to the final manuscript.

## Conflict of Interest

The authors declare that the research was conducted in the absence of any commercial or financial relationships that could be construed as a potential conflict of interest.

## Abbreviations

PCa: prostate cancer
RP: radical prostatectomy
IMRT: intensity modulated radiotherapy
BT: brachytherapy
EWGSOP: European Working Group on Sarcopenia in Older People
ACM: all-cause mortality
CCM: cancer-specific mortality
MRI: magnetic resonance imaging
CCI: Charlson comorbidity index
ECOG: Eastern Cooperative Oncology Group
ASA: American Association of Anesthesiologists
SMI: skeletal muscle index
OS: overall survival
CT: computed tomography
HU: Hounsfield unit
BMI: body mass index
HR: hazard ratio
CI: confidence interval.
